# Perspective on Clinical and Functional Outcomes of Arthroscopic All-Inside Meniscal Repair: Insights From a Lower Middle-Income Country

**DOI:** 10.7759/cureus.62664

**Published:** 2024-06-19

**Authors:** Rufina Ali, Muhammad Ahsan Sulaiman, Fizzah Mariam, Naveed Baloch

**Affiliations:** 1 Surgery/Orthopedics, Aga Khan University Hospital, Karachi, PAK

**Keywords:** arthroscopic repair, meniscal repair, lysholm score, lower-middle economic country, arthroscopic all inside meniscal repair

## Abstract

Background

Meniscal tears are a common injury in the adult population. With the advent of newer devices, the adoption of the all-inside repair technique has been expanding substantially because of its feasibility and reduced risk to surrounding neurovascular structures. This study was conducted in a lower middle-income country to assess the functional outcome of the arthroscopic all-inside technique and to identify the potential factors that may affect the functional outcome that will eventually influence the future management of these patients.

Method

This study is a retrospective case series conducted at the Department of Orthopedic Surgery, Aga Khan University Hospital, Karachi, Pakistan. Patients presenting to the outpatient clinics with meniscal tears who underwent arthroscopic all-inside repair from January 2015 to December 2021 were included in this study. The exclusion criteria included patients who had associated fractures and patients with meniscal tears greater than six months ago.

Results

A total of 29 patients underwent all-inside meniscal repair for meniscus tears. The mean age of our patients was 26.31 years (SD = 7.11 years), ranging from 17 years to 48 years. Of these patients, 26 were males and three were females, accounting for 89.7% and 10.3%, respectively. The most frequent mechanism of injury was twisting while playing sports, accounting for 51.7%, followed by falling while playing sports and road traffic accidents (RTAs), accounting for 13.8% and 20.7%, respectively. Of the 29 patients, 16 (55.2%) had lateral meniscal injuries, 10 (34.5%) were diagnosed with medial meniscus injuries, and three (10.3%) had injuries to both menisci. The most common type of tear that was observed in our sample size was bucket handle tears, which were found in 14 patients, accounting for a total of 48.3%, followed by complex tears in seven patients (24.1%). The majority of the patients, i.e., 19 out of 29 patients (65.5%), had an acute course of injury, i.e., less than six weeks. For the functional outcome, the Lysholm score was calculated at 12 months and was found to be excellent in 17 patients, good in six patients, and fair in six patients, accounting for 58.6%, 20.7%, and 20.7%, respectively. The mean Lysholm score was 90.03 ± 8.85 points. Of the 29 patients, 27 (93.2%) had no complaints at the regular 12-month follow-up, whereas one patient (3.4%) experienced rotatory instability and one patient (3.4%) experienced stiffness at the knee joint. None of the patients had to undergo a reoperation. The mean Lysholm score in the 25 patients who had an associated anterior cruciate ligament tear was 89.64 ± 9.442 points, whereas the four patients who had an isolated meniscal tear had a mean score of 92.50 ± 2.887 points, which was not significantly different (p-value = 0.831).

Conclusion

All-inside meniscal repair for treating meniscal tears has become the new treatment paradigm as it not only renders excellent functional outcomes with minimal complications but also prevents damage to the surrounding neurovasculature and the soft tissue envelope as it is a minimally invasive technique.

## Introduction

The menisci are the crucial fibrocartilaginous structures on both sides (medial and lateral) of a knee, and they play intricate biomechanical roles in stabilizing the knee joint and providing a cushioning effect. Both menisci bear 40-70% of the weight across the knee and distribute the load, thereby reducing the stresses on the tibia and maintaining the articular congruency of the knee joint [1,2]. The incidence rate of meniscal tears in the general population is estimated to be 60 per 100,000 meniscal injuries, with males being affected more than females [3]. The occurrence of meniscal injuries has a bimodal age distribution; in the younger population, meniscal injuries occur as a consequence of any acute trauma, while degenerative changes are more likely to occur in old age [4]. Meniscal injuries frequently occur with concomitant anterior cruciate ligament (ACL) tears, with an incidence rate of up to 82% and 96% in acutely injured ACLs and chronically deficient ACLs, respectively [5]. Due to its mobility and fewer attachments, the lateral meniscus is less susceptible to tearing, whereas the medial meniscus is less mobile than the lateral meniscus; therefore, the lateral meniscus is more prone to injuries [6]. A wide variety of symptoms, including pain that is localized to the joint line, swelling, clicking, and locking of the knee, can be caused by meniscal tears [7]. The preferred imaging modality for assessing menisci and diagnosing meniscal tears is MRI [8].

Conventionally, the main treatment modality for meniscal tears is meniscectomy (open or arthroscopic, partial or total), but in recent years, meniscal repair has gained immense popularity, and meniscectomy has been avoided whenever possible. The reasons for this shift in the choice of treatment are the negative consequences of meniscal resection, most notably the onset of early osteoarthritis and poor long-term clinical outcomes, thus affecting quality of life [9]. The repair techniques aim mainly at the preservation of the remaining menisci. Meniscal repair techniques must be tailored according to the type, size, and location of the tear, the mechanism of injury, and patient-related factors such as age, lifestyle, and general health status. Currently, there are three principal techniques, namely, inside out, outside inside, and all inside, and all three of these surgical modalities have their own merits and demerits [10]. The inside-out technique has been considered the gold standard for meniscal repair, although it can be time-consuming and is associated with damage to the neurovasculature, thereby increasing morbidity [11,12] With the advent of newer devices, the adoption of all-inside repair techniques has substantially expanded because of their feasibility and reduced risk to surrounding neurovascular structures [13,14]. A systematic review by Vint et al. revealed no difference in the functional outcomes between the two techniques; however, the operative time is shorter for the all-inside technique of meniscal repair [15]. Novel methodologies for orthopedic procedures are still being introduced to developing nations despite multiple obstacles, such as resource constraints in terms of financial, educational, and expertise [16].

To measure the functional outcome of patients with knee injuries, a 100-point scoring system known as the Lysholm knee scoring system, which consists of eight components, namely, a limp while walking, a cane or crutches while walking, a locking sensation, a giving away sensation, pain, swelling, problems while climbing stairs, and problems in squatting, is utilized [17].

We investigated the clinical and functional outcomes of arthroscopic all-inside meniscal repair in an adult population who presented to a tertiary care center in a lower middle-income country.

## Materials and methods

This retrospective case series was conducted at the Department of Orthopedic Surgery, Aga Khan University Hospital, Karachi, Pakistan. Appropriate approval was obtained from the ethical review committee of the hospital before the commencement of this study (approval number 2022-8035-22952).

Patients presenting to the outpatient clinics with meniscal tears who underwent arthroscopic all-inside repair from January 2015 to December 2021 were included in this study. The exclusion criteria included patients who had associated fractures and patients with meniscal tears older than six months.

All patients with meniscal injuries with or without ACL injuries were booked from the clinic for daycare surgery. The diagnosis was made on the basis of clinical and radiological findings such as locking symptoms, a McMurray test, and an MRI scan. Pre-anesthesia fitness was taken as routine protocol. General anesthesia was given to all patients. The procedure was done as a daycare or a single-day hospital stay, as per the patient’s preference.

**Figure 1:**
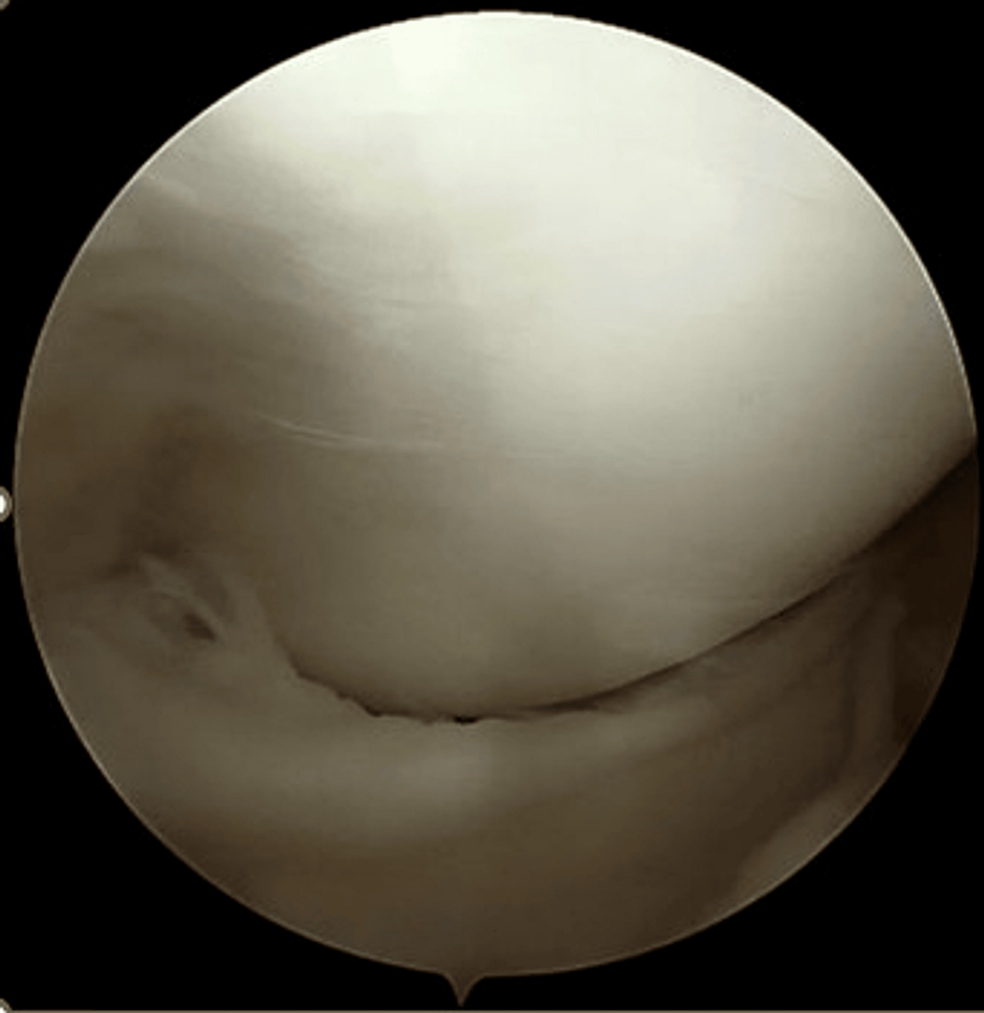
Initial presentation of the joint during diagnostic arthroscopy

**Figure 2:**
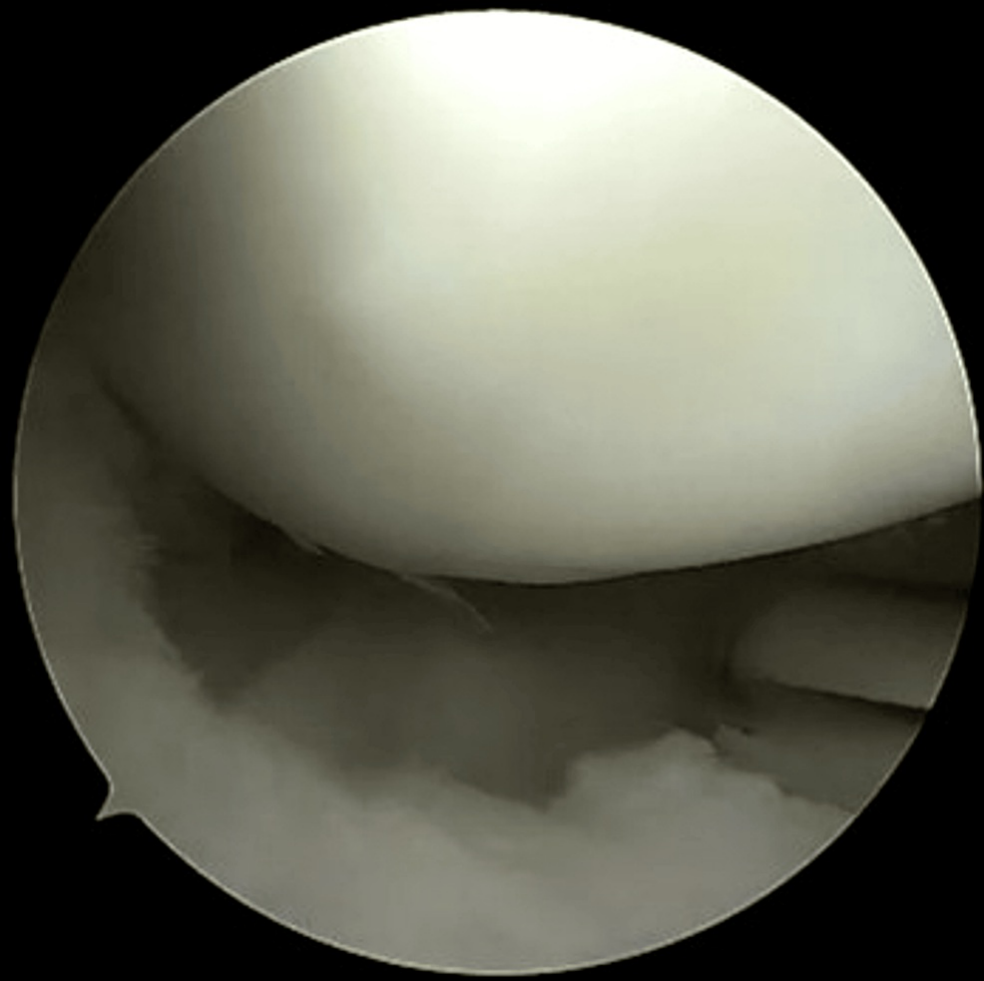
Bucket handle tear identification and reducibility assessment

**Figure 3:**
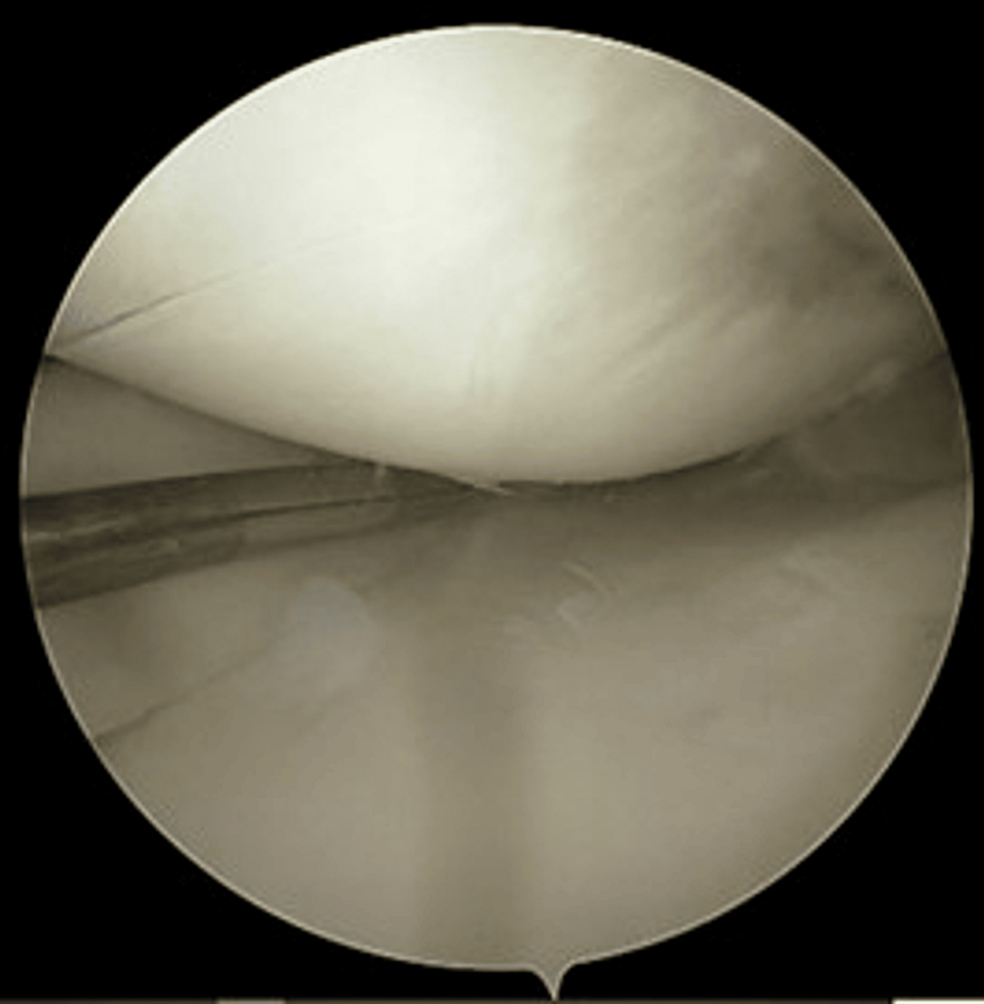
Debridement behind and at the periphery of the meniscus using a 3.2-mm arthroscopic shaver

**Figure 4:**
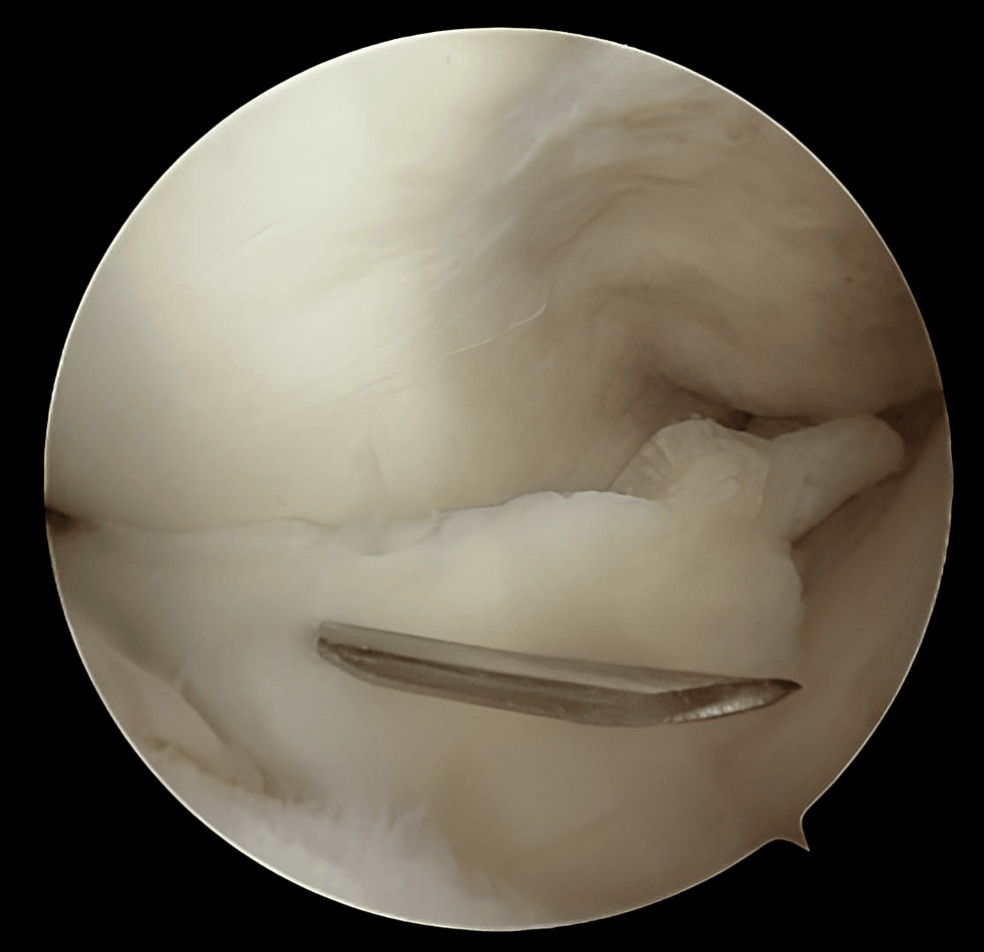
Meniscus reduction assisted by a needle

**Figure 5:**
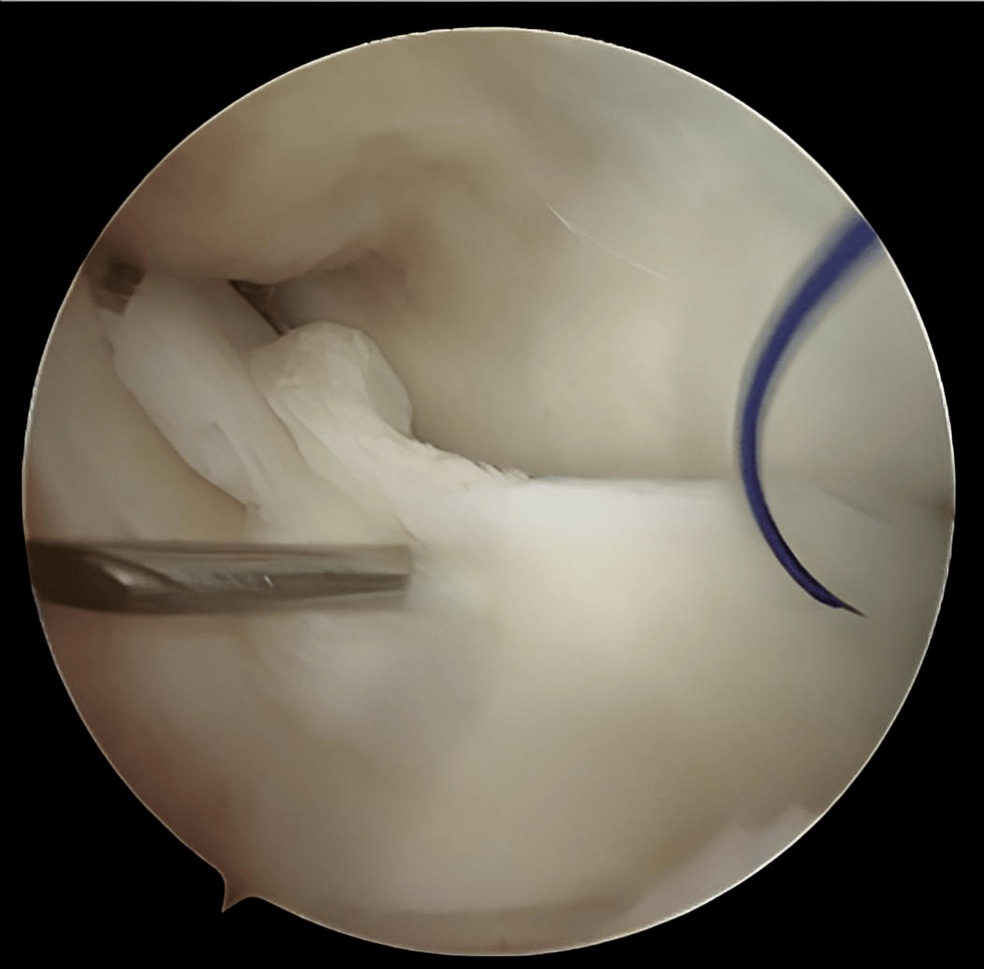
Anterior margin suture placement using an outside-in Prolene suture to maintain reduction

**Figure 6:**
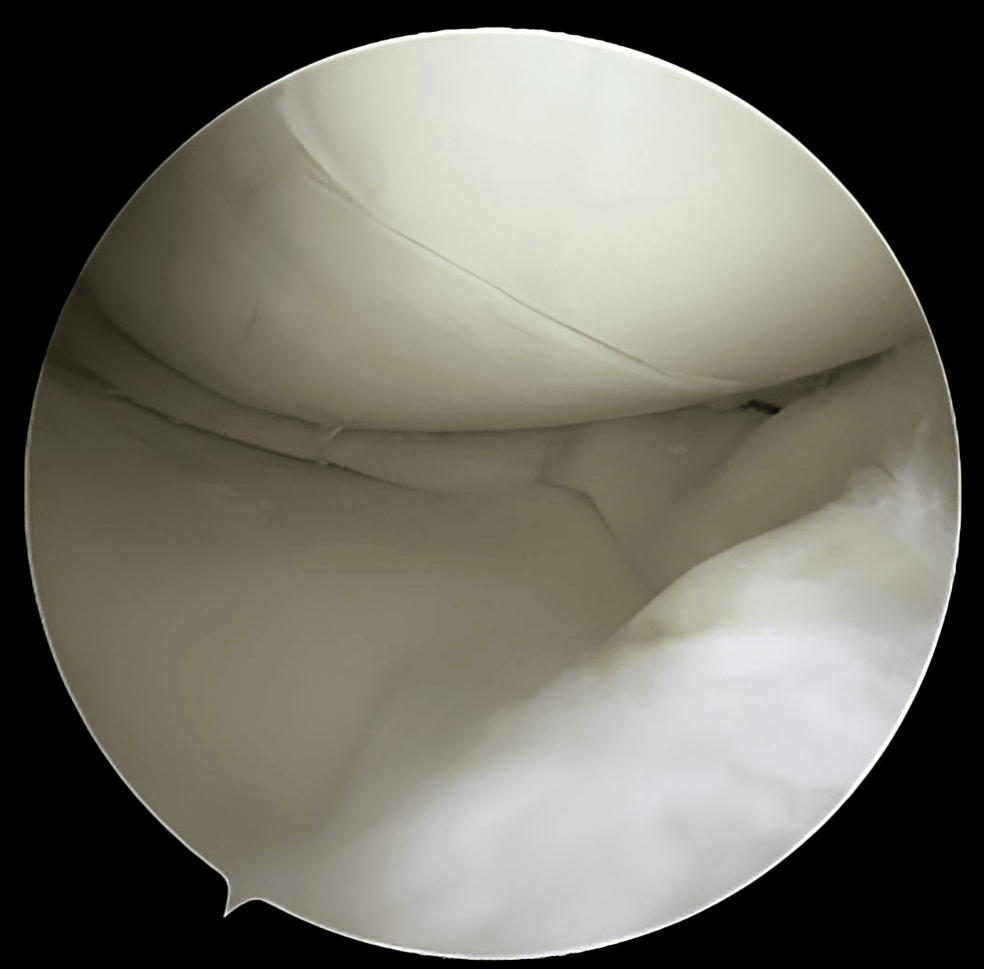
Outside-in suture to maintain the reduction of the meniscus

**Figure 7:**
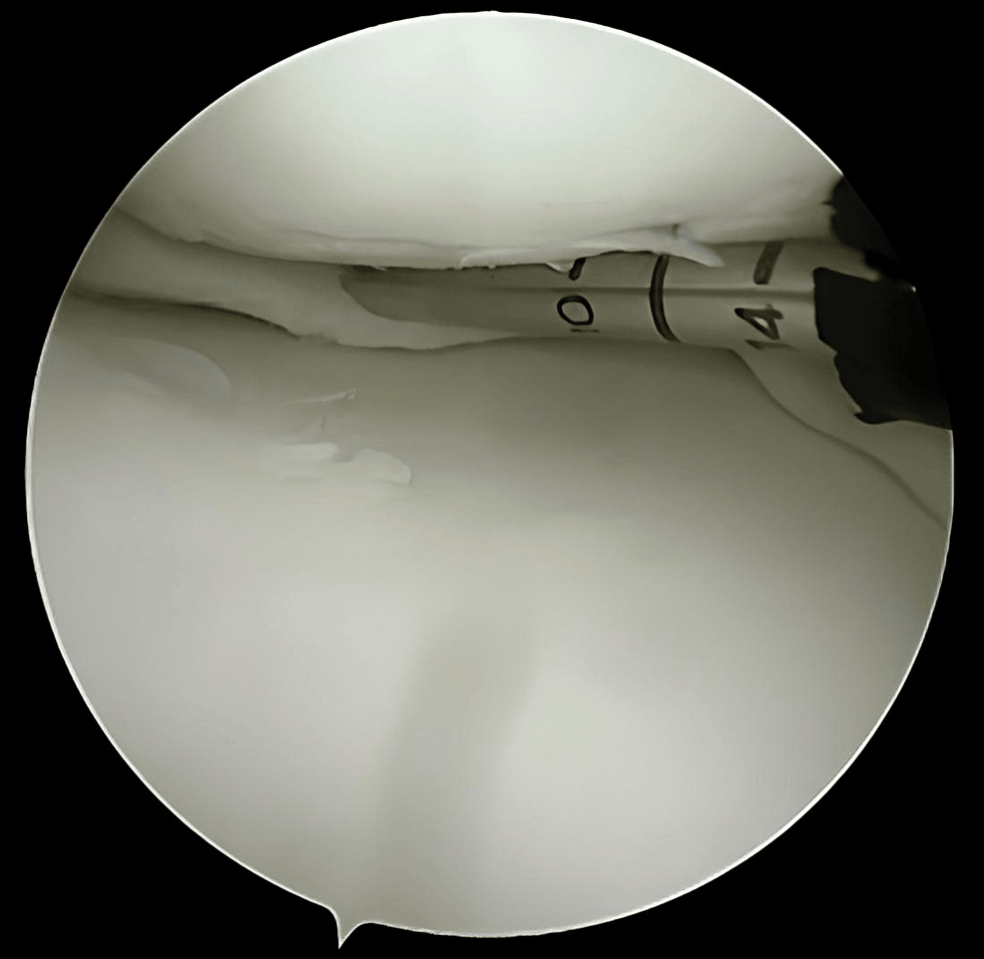
Appropriate length was taken (deploy with Arthrex All Inside Cinch™ was used)

**Figure 8:**
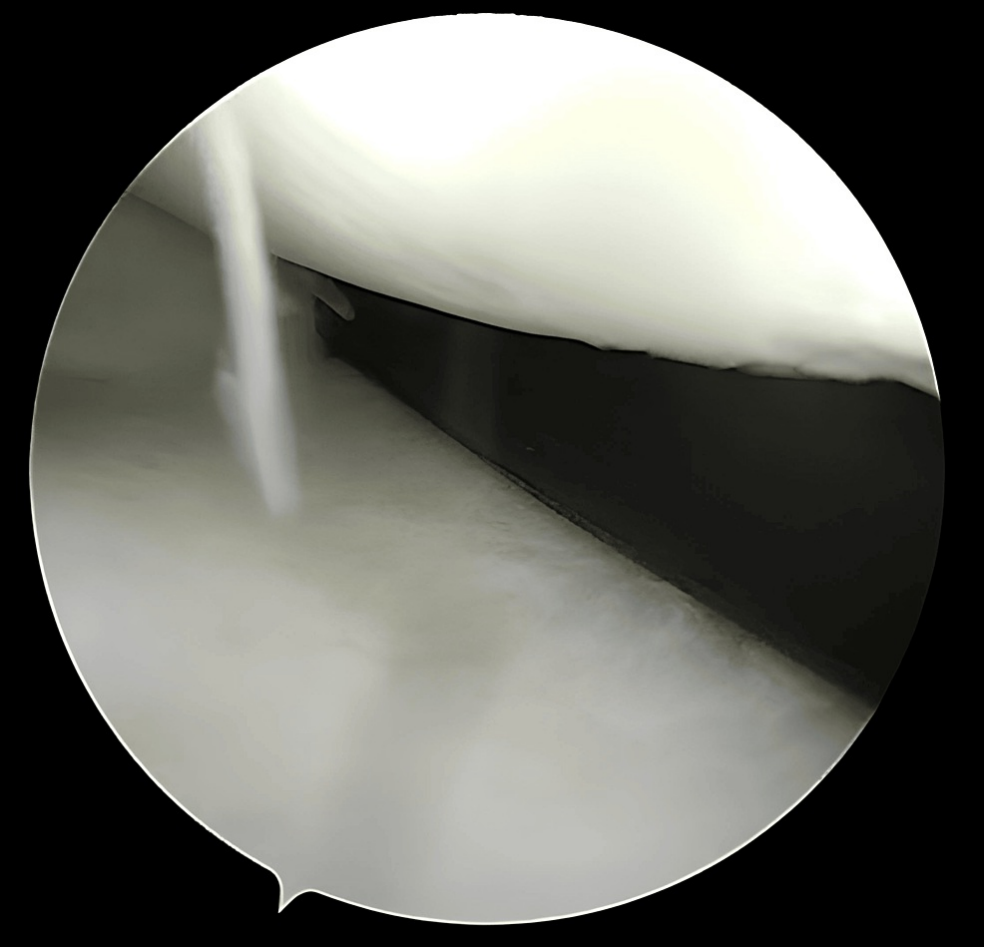
The needle is positioned over the desired entry point prior to deploying the suture

**Figure 9:**
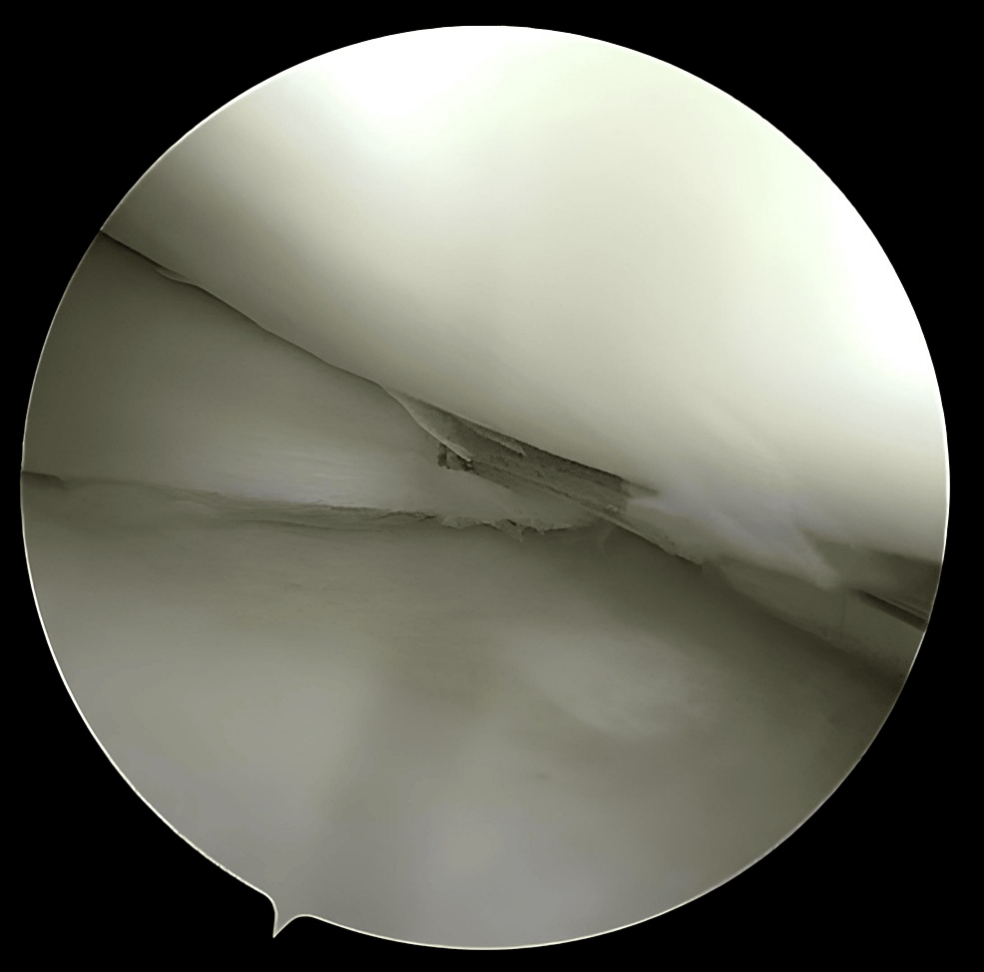
Complete deployment of the first pass using Arthrex All Inside Cinch™ for meniscus repair

**Figure 10:**
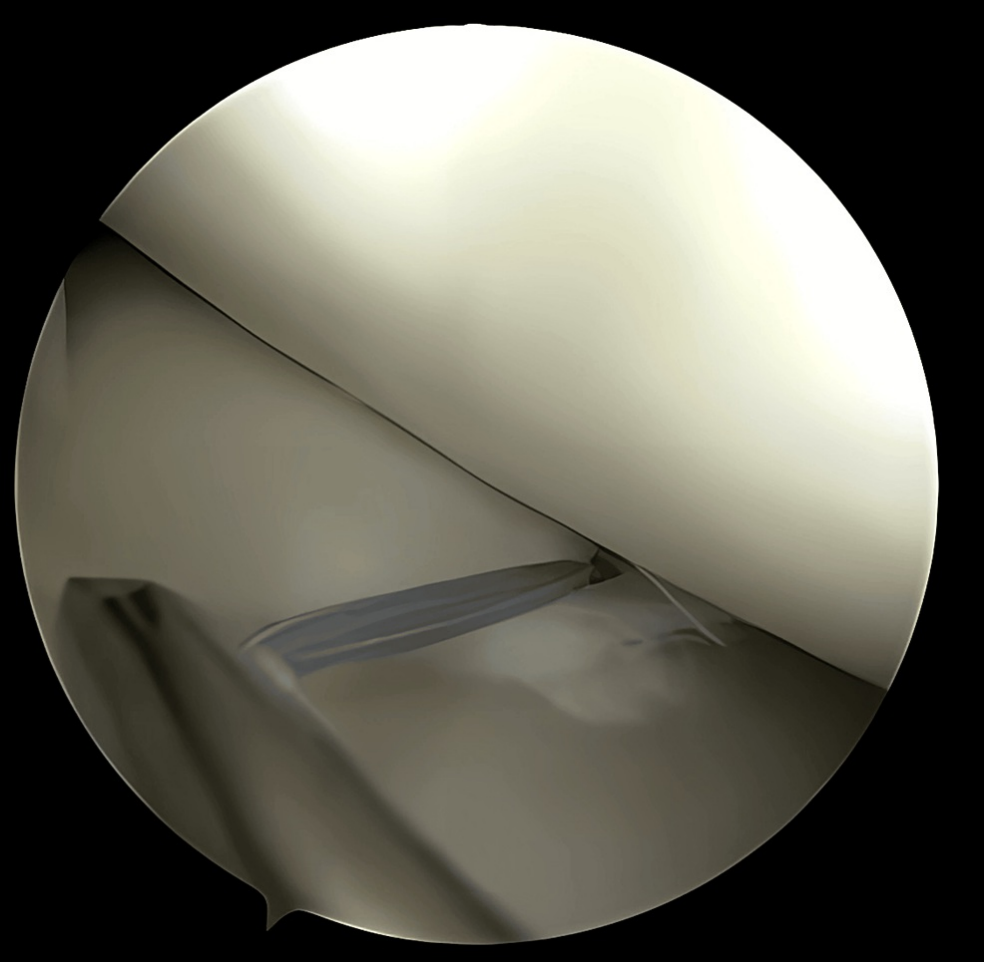
Repositioning the arthroscope to note the desired position for the second pass

**Figure 11:**
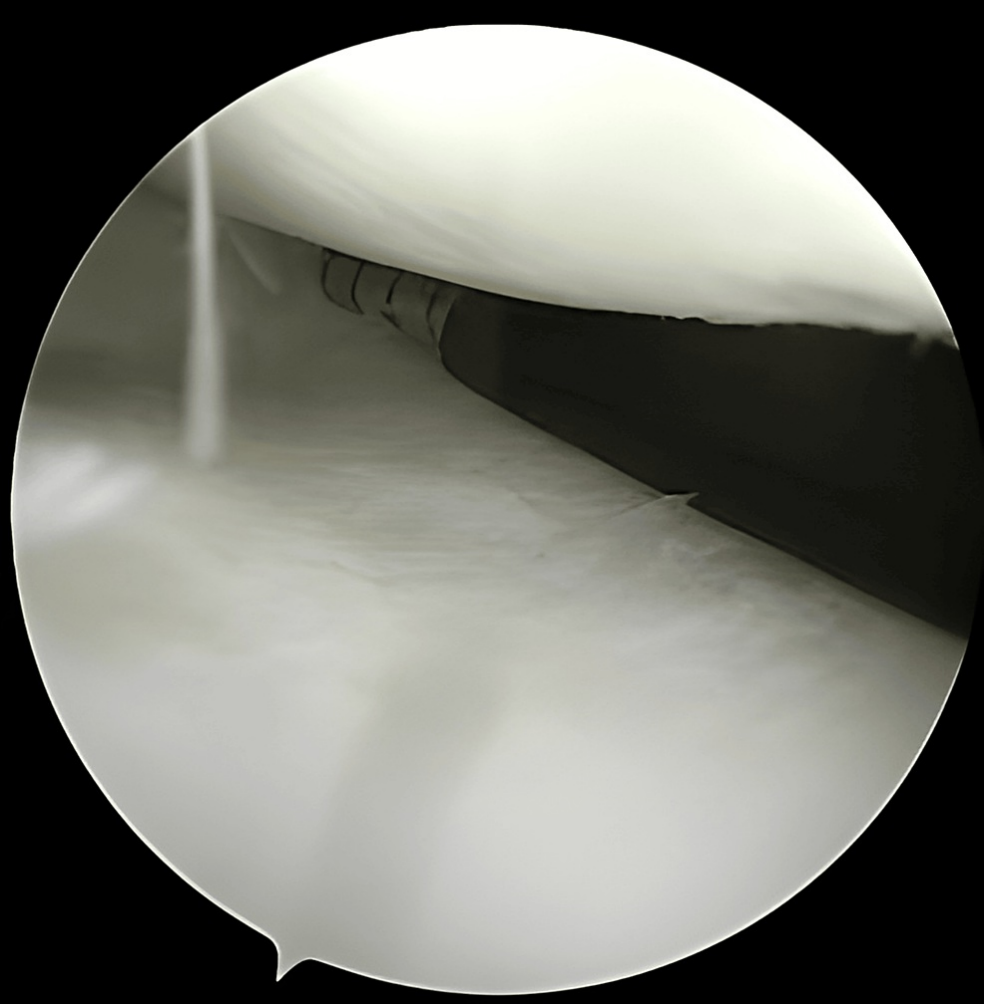
Measuring the appropriate depth for the second pass

**Figure 12:**
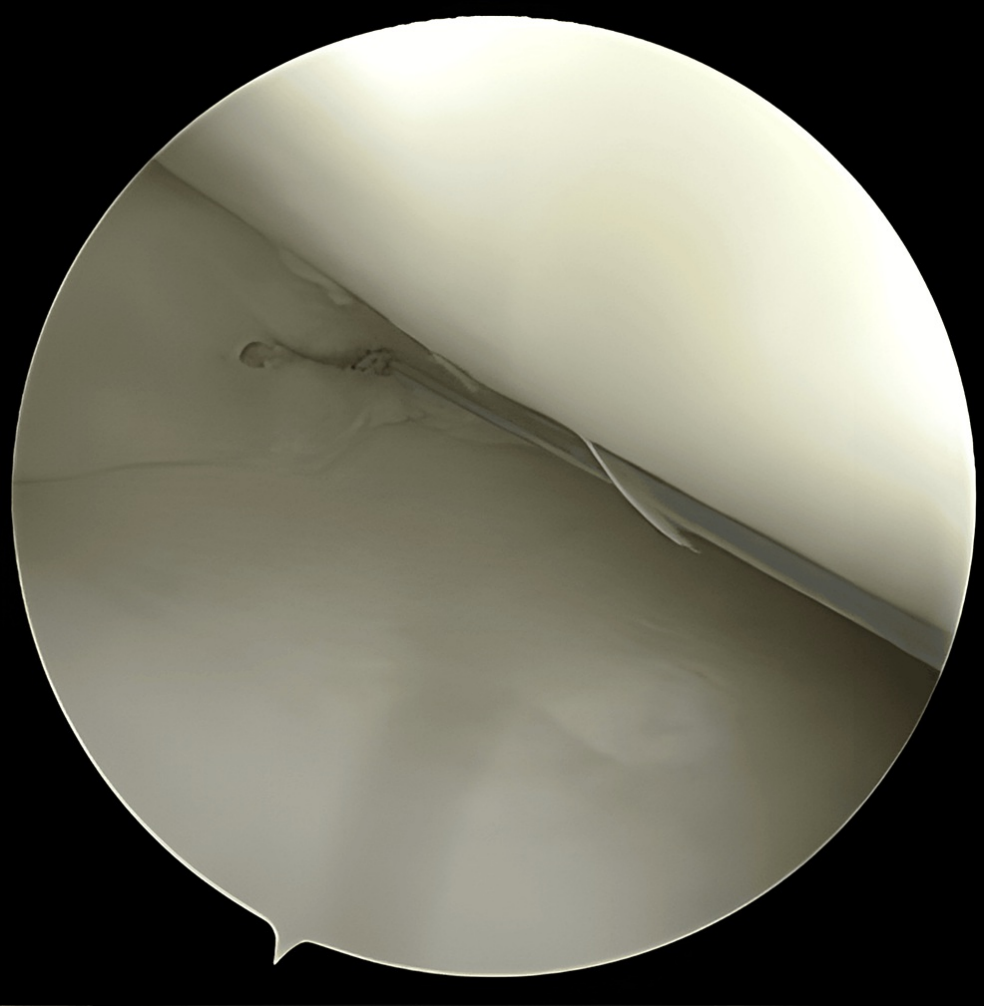
Complete deployment and advancement of the second pass After selecting the final position, the second pass is deployed using Arthrex All Inside Cinch™, advanced with a knot pusher, and excess suture is cut.

**Figure 13:**
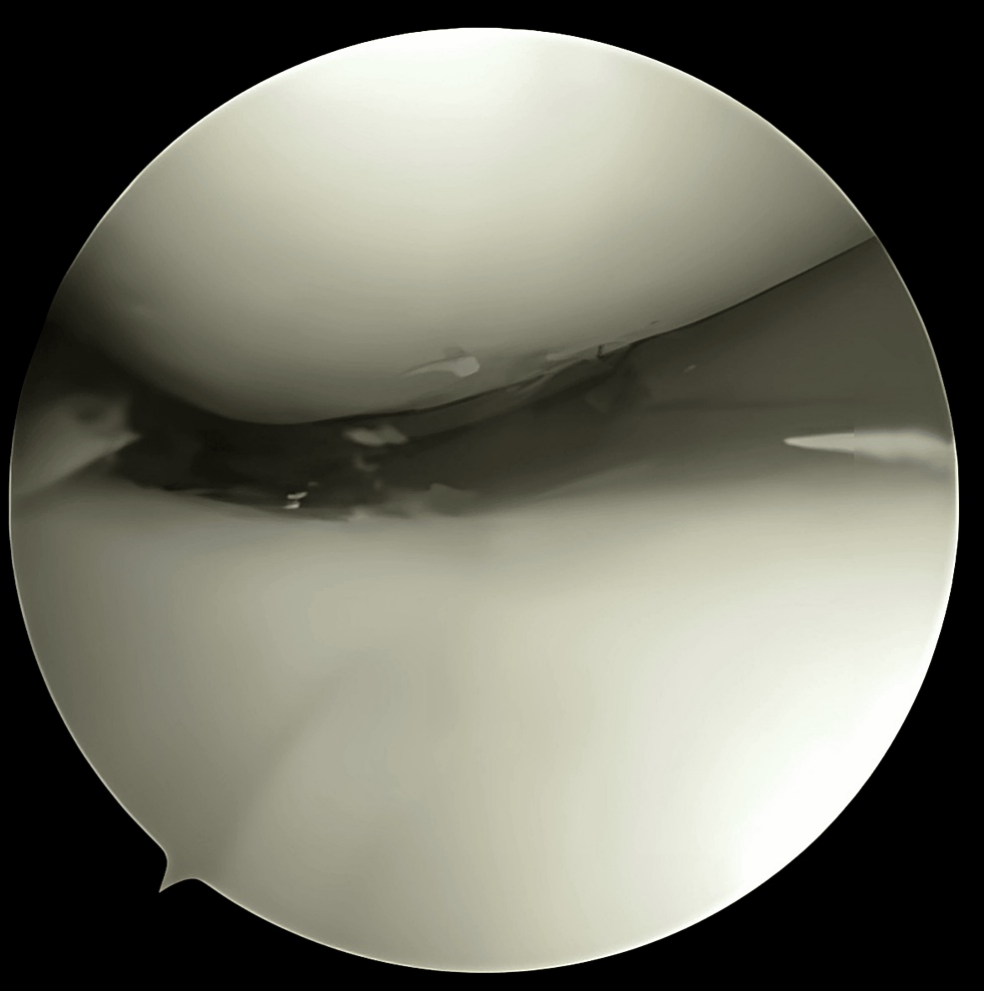
Final result at the end of meniscal repair

For the procedure, the patient was placed in a supine position with the involved leg near the edge of the table. The lead surgeon does knee arthroscopy in a sitting position on a rotating stool at the side of the table with the foot of the patient’s leg placed in the lap. No valgus support or any bolster under the hip is used. Standard portals were marked with the knee in 90 degrees of flexion. A pneumatic tourniquet was placed at the proximal thigh and inflated at 300 mmHg. The anterolateral portal was made for diagnostic arthroscopy, followed by an anteromedial portal under direct vision (Figure 1). A meniscus tear was identified, and an assessment of reducibility was done (Figure 2). Initial debridement from the periphery and behind the meniscus was done with the help of a 3.2-mm arthroscopic shaver (Figure 3). After reduction, an outside suture was used at the first anterior margin of the tear, which helps in maintaining reduction (Figures 4-6). For all-inside techniques deployed, Arthrex All Inside Cinch™ (Arthrex, Inc., Naples, United States) was used. The appropriate length was taken through Cinch, and the needle was placed over the desired entry point (Figure 7). The first pass was deployed completely, the second pass position was noted, and the appropriate depth was measured (Figures 8-10). The second pass was deployed completely, advanced via knot pusher, and excess suture was cut (Figures 11-13). The portal was closed with a 3/0 ethilon suture. An aseptic dressing was done. After recovery from anesthesia, the patient was reassessed for any anesthesia complications and discharged. If any of the patients had concomitant ACL tears, they underwent routine arthroscopic ACL reconstruction with a hamstring graft. The average duration for each meniscal surgery was 90 minutes.

Postoperatively, the knee was kept in full extension in a knee immobilizer, and the patient was taught by the primary surgeon to ambulate non-weight-bearing on their operated leg with walker support. On the first follow-up, three days later, the knee immobilizer was removed and the knee was flexed to 90 degrees. Isometric quadriceps and knee range of motion exercises were taught by the operating surgeon, and these exercises, non-weight bearing status with a knee immobilizer, and intermittent 90 degrees of knee flexion were continued for six weeks. After that, formal physiotherapy was taken, and board and full-weight bearing with walker ambulation started.

Data were collected by reviewing the medical records of all the recruited patients by a dedicated orthopedic research associate. The variables of interest that we extracted were related to patient demographics, including age, sex, and occupation, or to the injury, including the mechanism of injury, site of injury, type of meniscal tear, chronicity (acute tear from symptoms of pain until six weeks, chronic after six weeks), and association with ACL injury. Patients were followed at routine clinical visits for three, six, and 12 months, and functional outcomes were recorded by calculating the Lysholm knee score at the end of 12 months. All the patients were assessed for any postprocedural complications, including infection, stiffness, or the need to undergo redo surgery.

After the data were collected, they were compiled and analyzed using IBM SPSS Statistics for Windows, Version 23.0 (Released 2015; IBM Corp., Armonk, NY, USA). A p-value of <0.05 was considered significant throughout the study. Quantitative variables such as age are reported as the mean and standard deviation. Qualitative variables such as sex and complications are presented as frequencies and percentages.

## Results

A total of 29 patients underwent all-inside meniscal repair for meniscus tears. The mean age of our patients was 26.31 years (SD = 7.11 years), ranging from 17 years to 48 years. Of these patients, 26 were males and three were females, accounting for 89.7% and 10.3%, respectively. The most frequent mechanism of injury was twisting while playing sports (football and volleyball), accounting for 15 (51.7%) patients, followed by falling while playing sports and RTAs accounting for six (20.7%) and eight (27.6%) patients, respectively, as shown in Table 1.

**Table 1:** Results obtained in the study ACL, anterior cruciate ligament; RTA, road traffic accident

Parameters		Results, n
Gender	Male	26 (89.7)
	Female	3 (10.3)
Mechanism of injury	Twisting (sports)	(51.7%)
	Fall	(13.8%)
	RTA	-20.70%
Timings	Acute (<6 weeks)	19 (65.5%)
	Chronic (>6 weeks)	10 (34.5%)
Site	Lateral meniscus	16 (55.2%)
	Medial meniscus	10 (34.5%)
	Both	3 (10.3%)
Types of tear	Bucket handle	14 (48.3%)
	Complex	7 (24.1%)
	Horizontal	4 (13.8%)
	Radial	4 (13.8%)
Concomitant ACL tear	Yes	25 (86.2%)
	No	4 (13.7%)

Of the 29 patients, 16 (55.2%) had lateral meniscal injuries, 10 (34.5%) were diagnosed with medial meniscus injuries, and three (10.3%) had injuries to both menisci. The most common type of tear that was observed in our sample size was bucket handle tears, which were found in 14 patients, accounting for a total of 48.3%, followed by complex tears in seven patients (24.1%). The majority of the patients, i.e., 19 out of 29 patients (65.5%), had an acute course of injury, i.e., <6 weeks, as shown in Table 1. None of the patients presented with a bilateral knee meniscal tear.

Out of 29, 25 (86.2%) patients had a concomitant ACL tear along with a meniscus tear, whereas only four (13.7%) had isolated meniscal injuries.

For the functional outcome, the Lysholm score was calculated at 12 months and was found to be excellent in 17 patients, good in six patients, and fair in six patients, accounting for 58.6%, 20.7%, and 20.7%, respectively. The mean Lysholm score was 90.03 ± 8.85 points. Of the 29 patients, 27 (93.2%) had no complaints at the regular 12-month follow-up, whereas one patient (3.4%) experienced rotatory instability and one patient (3.4%) experienced stiffness at the knee joint. None of the patients had to undergo a reoperation. The mean Lysholm score in the 25 patients who had an associated ACL tear was 89.64 ± 9.442 points, whereas the four patients who had an isolated meniscal tear had a mean score of 92.50 ± 2.887 points, which was not significantly different (p-value = 0.831) (Table 2).

**Table 2:** Lysholm Knee Score

Lysholm Knee Score		Number of patients
Excellent	95-100	17 (58.6%)
Good	84-94	6 (20.7%)
Fair	65-83	6 (20.7%)
Poor	Less than 64	0 (0%)

## Discussion

The all-inside technique for meniscal repair has recently gained popularity worldwide because it is not only less demanding for surgeons but also has a shorter duration of surgery than other techniques for meniscal repair, and it also seems to cause fewer postoperative complications while being a bit more expensive than the traditional meniscal repair with Prolene sutures [4,13]. The all-inside technique is, although more expensive, compared to the other techniques for meniscal repair. There are no accessory incisions required for this surgical method, which in turn avoids the surgical risk of neurovascular damage [18]. The sole aim of this study was to determine the clinical outcome and occurrence of complications after the use of all-inside meniscal repair techniques in a lower middle-income country. In a systematic review by Grant et al., it was concluded that the all-inside technique is associated with implant-related complications and soft tissue injury postoperatively [11]. However, according to our findings, stiffness at the knee joint and rotatory instability were the only complications that were encountered. This could be due to advanced age and not following the physiotherapy protocol postoperatively.

The resolution of clinical symptoms, i.e., locking, swelling, or joint pain, only provides indirect evidence of successful healing, and the current literature has no accepted definition of failure of meniscal repair [18,19]; however, according to Noyes and Barber-Westin, failure of repair can be defined as the nonresolution of clinical symptoms and/or the need for repeat meniscectomy and arthroscopy [20]. The success rate in our study was 93.2%, which is comparable to that in the previous literature [11,21-23]. As a lower middle-income nation, the majority of the populace lacks insurance coverage and has to personally bear the financial burden. High success rates enhance the efficacy of these techniques in mitigating the need for reoperation and, in turn, benefit patients. For the clinical evaluation, the Lysholm score [16] was calculated, and the mean score was 90.03 points, which indicates that the treatment paradigm of all-inside meniscal repair renders a clinically successful outcome in a majority of cases. In a study by Hoffelner et al., the mean Lysholm score was 76 points [21]. Martin-Fuentes et al. reported a mean Lysholm score of 88 with a clinical success rate of 83% [24]; however, the follow-up timeline in previous studies was longer than that in the present study, as our follow-up was until 12 months.

Previous literature suggests that the healing rate is significantly better when ACL reconstruction is performed concurrently with meniscal repair, which could be attributed to the fact that fibrin clots are formed during ACL reconstruction and postoperative hemarthrosis creates a favorable environment for healing [18,19]. However, many studies have contradicted this finding, with results similar to those of the present study [24-26]. In our study, we observed consistently good clinical results with meniscal repairs using an all-inside technique for isolated meniscal injuries and for meniscal tears associated with ACL reconstruction, with Lysholm scores of 92.50 and 89.64, respectively (p-value = 0.831). Martin-Fuentes et al. reported similar clinical outcomes for knees treated with and without concomitant ACL reconstruction, with Lysholm scores of 88 and 88.6 points, respectively [24]. Laurendon et al. [26] reported similar results, with no significant difference between intact and reconstructed ACLs (P = 0.17).

The success rate of the inside technique for meniscal repair varies from 70% to 94% [26], and it remains a reliable technique with an expected success rate of >80% [25].

The present study is limited by its retrospective design, the small sample size, and the short follow-up period. The strengths of our study are that we included all consecutive patients in our series, and no patients were lost to follow-up.

## Conclusions

All-inside meniscal repair for treating meniscal tears with or without concomitant ACL injuries has become a new treatment paradigm because it not only results in excellent functional outcomes with minimal complications but also prevents damage to the surrounding neurovasculature and soft tissue envelope, as it is a minimally invasive technique. Our study also shows promising results for the all-inside meniscal repair.
